# The impact of preparatory activities on medical school selection outcomes: a cross-sectional survey of applicants to the university of Adelaide medical school in 2007

**DOI:** 10.1186/1472-6920-13-159

**Published:** 2013-12-01

**Authors:** Caroline O Laurence, Ian T Zajac, Michelle Lorimer, Deborah A Turnbull, Karen E Sumner

**Affiliations:** 1 Discipline of General Practice, School of Population Health, The University of Adelaide, North Terrace, Adelaide, South Australia 5005, Australia; 2 Commonwealth Scientific and Industrial Research Organisation, Preventative Health Flagship, Kintore Avenue, Adelaide, South Australia 5005, Australia; 3 School of Psychology, University of Adelaide, North Terrace, Adelaide, South Australia 5005, Australia; 4 Rural Doctors Workforce Agency, 63 Henley Beach Road, Mile End, South Australia 5031, Australia

**Keywords:** Medical school selection, Admissions, Equity, Preparation

## Abstract

**Background:**

Selection into medical school is highly competitive with more applicants than places. Little is known about the preparation that applicants undertake for this high stakes process. The study aims to determine what preparatory activities applicants undertake and what difficulties they encounter for each stage of the application process to medical school and in particular what impact these have on the outcome.

**Methods:**

A cross-sectional survey of 1097 applicants who applied for a place in the University of Adelaide Medical School in 2007 and participated in the UMAT (Undergraduate Medicine and Health Sciences Admission Test) and oral assessment components of the selection process. The main outcome measures were an offer of an interview and offer of a place in the medical school and were analysed using logistic regression.

**Results:**

The odds of a successful outcome increased with each additional preparatory activity undertaken for the UMAT (odds ratio 1.22, 95% confidence interval 1.11 to 1.33; P < 0.001) and the oral assessment (1.36, 1.19 to 1.55; P < 0.001) stage of selection. The UMAT preparatory activities associated with the offer of an interview were attendance of a training course by a private organisation (1.75, 1.35 to 2.27: P < 0.001), use of online services of a private organisation (1.58, 1.23 to 2.04; P < 0.001), and familiarising oneself with the process (1.52, 1.15 to 2.00; p = 0.021). The oral assessment activities associated with an offer of a place included refining and learning a personal resume (9.73, 2.97 to 31.88; P < 0.001) and learning about the course structure (2.05, 1.29 to 3.26; P = 0.022).

For the UMAT, applicants who found difficulties with learning for this type of test (0.47, 0.35 to 0.63: P < 0.001), with the timing of UMAT in terms of school exams (0.48, 0.5 to 0.66; P < 0.001) and with the inability to convey personal skills with the UMAT (0.67, 0.52 to 0.86; P = 0.026) were significantly less likely to be offered an interview.

**Conclusions:**

Medical schools make an enormous effort to undertake a selection process that is fair and equitable and which selects students most appropriate for medical school and the course they provide. Our results indicate that performance in the selection processes can be improved by training. However, if these preparatory activities may be limited to those who can access them, the playing field is not even and increasing equity of access to medical schools will not be achieved.

## Background

The selection of students into medical courses is a major issue for university medical schools and it is often embroiled in controversy [1-3]. With more applicants than places, the selection process is very competitive and often scrutinised to ensure that it selects applicants with suitable characteristics for medicine and no systemic biases exist within the process. In countries such as Australia [4], NZ [5], Canada [6], US [6] and the UK [7] the selection process to medical school consists of an aptitude/cognitive test, non-cognitive measures such as interviews and ratings of academic achievement such as matriculation score or grade point average.

While a variation on these processes is widely used, the evidence base for selection is small [8]. Most research has focused on the predictive validity of different parts of the selection process, such as the aptitude/cognitive tests like the MCAT [9,10], UKCAT [11-13], or UMAT [14,15] or ratings of academic achievement such as grade point average [16].

Another small body of research has focused on demographic and socio-economic factors that may influence or predict the outcome of the process and if the process biases against disadvantaged groups [17-20].

There is little research regarding the lengths applicants go to in order to maximise their chance of receiving an offer or the difficulties they encounter during this process [21-23]. Moreover, there is little evidence on of the impact of the preparatory activities undertaken or difficulties encountered on the outcome [24]. The aims of this study are: to determine what preparation applicants undertake for each stage of the application process at the University of Adelaide Medical School (UMAT and oral assessment); to determine what difficulties they encounter at each stage of the application process; and if the preparatory activities or difficulties encountered impact on the outcome of the selection process.

## Methods

### Survey

We conducted a questionnaire-based survey of all applicants who applied to the University of Adelaide for entry into the undergraduate medicine course in 2007. A summary of the selection process at the University of Adelaide Medical School is provided in Table 1. The selection process has three parts. Firstly, applicants sit the Undergraduate Medical and Health Sciences Admission Test (UMAT) and their score for this test determines who proceeds on to the next stage of the process, the oral assessment. Those who undertake the oral assessment receive a score. An offer of a place is then based on a composite score of the UMAT, oral assessment score and an applicant’s Australian Tertiary Admissions Rank. A student who receives an offer can either accept the place or not.

**Table 1:** The selection process at the University of Adelaide at time of study

**Application stage**	**Description**	**Assessing**	**Applicant no.s**
1. UMAT (Undergraduate medicine & health sciences admission test)	Managed nationally by Australian Council for Educational Research (ACER)	● Logical reasoning and problem solving	>2500
	Test held mid year, prior to the December selection process	● Understanding	
	Written examination	● people	
	No preparation required Used as a screening tool	● Non-verbal reasoning	
2. Oral assessment	Structured interview (35 minutes)	● Non-cognitive qualities	~350
	Two assessors – Faculty & community	● Humanistic qualities	
3. ATAR (Australian Tertiary Admissions Rank)	Score of 90 or above (max score 100)	● Academic ability	190

International students applying for fee-paying places were excluded as the selection process varied slightly for this group. The survey consisted of four questionnaires which corresponded with the various stages of the application process – applicants who participated in the UMAT, applicants who were offered an oral assessment, applicants who were offered a place but did not accept and applicants who were offered a place and accepted. For each successive group an additional section was included that pertained to the particular stage in the application process. The questionnaires were developed following a focus group with first year medical school students who had experienced the application process approximately six months earlier and a review of the literature. The questionnaires covered a number of areas including socio-demographics, interest in medicine, preparatory activities and difficulties encountered during the various stages of the application process. A copy of the key components of the questionnaire used in this analysis are provided Additional file 1.

The questionnaires were mailed to applicants between March and June 2007, following the completion of the medical school application process. In order to maximise the response rate the project team adopted Dillman’s Total Design Method [25]. This involves mailing out an initial questionnaire, which is followed by a reminder and after that a final questionnaire for those who do not respond.

### Statistical analysis

Chi-squared tests and two sample t-tests (where appropriate) were used to determine if there were differences between responders and non-responders in terms of age, sex and location of residence.

For the responders, three outcome groups were determined – those who completed the UMAT, those who attended the oral assessment and those who were offered a place, irrespective of whether they accepted the offer or not. The analysis was undertaken in two parts reflecting the different stages of the application process. Firstly, those offered an oral assessment interview following the UMAT were compared with those who were not offered an oral assessment interview. Then for those who progressed to the oral assessment, comparisons were then made between those who were offered a place with those who were not offered a place in the medical school.

The number of preparatory activities was calculated and logistic regression used to determine if there was an association between the number of preparatory activities undertaken and a successful outcome (offer of an interview or offer of a place).

To determine what preparatory activities (or difficulties) were undertaken, separate logistic regression analyses were performed for each outcome.

Statistical significance was set at 5%. To account for multiple testing, Bonferroni Correction was applied for each preparatory activity and difficulty encountered, resulting in a corrected P value. The corrected and uncorrected P values are reported in the tables, along with odds ratios and 95% confidence intervals. All analysis was performed using SAS 9.2 (SAS Institute, Cary NC, USA).

This study was approved by the University of Adelaide Human Research Ethics Committee.

## Results

A total of 2150 questionnaires were distributed and an overall response rate of 51% (1097/2150) was achieved. Response rates varied with the application stage. For applicants who did not receive an offer of an interview following the UMAT, the response rate was 46% (739/1610). For those applicants who participated in the oral interviews but did not receive and offer, the response rate was 55% (108/201), while a response rate of 74% (250/339) was obtained from those applicants who received an offer of a place.

A comparison of age and sex of responders to non-responders showed there were no statistical differences in the groups in terms of age with both groups having the same mean age (17.7 years, p = 0.68). However, significantly more females and fewer males were in the response group (60% and 40% respectively) than the non-response group (52%, 48% respectively, p <0.001).

### Participant characteristics

The socio-demographic characteristics of the respondents are summarised in Table 2. Of all applicants in the study, there were a greater proportion of female applicants (60%) and applicants who attended a non-government school (57%). Over a third of respondents were from South Australia (37%) and 78% spoke English as their main language. Over half (56%) of the respondents had a family member who worked in the health profession, with the most common family member being their mother (57%). 15% of respondents had lived rurally for at least eight or more years.

**Table 2:** Demographic characteristics of respondents by outcome

**Characteristics**		**Completed UMAT but not offered oral assessment (*N*** **= 738)**	**Completed, UMAT, offered oral assessment but were not offered a place (*N*** **= 109)**	**Completed UMAT and oral assessment and were offered a Place (*N*** **= 250)**	**All applicants in the study (*N*** **= 1097)**
Age in Years	Mean	18.0	17.9	17.9	18.0
	*(SD)*	*(1.7)*	*(0.7)*	*(0.9)*	*(1.4)*
	Median	*18.0*	*18.0*	*18.0*	*18.0*
	Range	15-49	16-19	17-23	15-49
Sex (%)	Male	295 (40.0)	45 (41.3)	102 (40.8)	442 (40.3)
	Female	443 (60.0)	64 (58.7)	148 (59.2)	655 (59.7)
Schooling (%)	Non-Government	407 (55.2)	63 (57.8)	154 (61.6)	624 (56.9)
	Government	268 (36.3)	43 (39.5)	86 (34.4)	397 (36.2)
	Other (eg overseas school)	22 (3.0)	2 (1.8)	5 (2.0)	29 (2.6)
	Combination of non-Governement and Government	36 (4.9)	1 (0.9)	5 (2.0)	42 (3.8)
Main language (%)	English	555 (75.2)	89 (81.7)	212 (84.8)	856 (78.0)
	Other	172 (23.3)	20 (18.4)	37 (9.7)	229 (20.9)
	Missing	11 (1.5)	0	1 (0.7)	12 (1.1)
Lived rurally (%)*	Yes	115 (15.6)	18 (16.5)	27 (10.8)	160 (14.6)
	No	623 (84.4)	91 (83.5)	223 (58.2)	937 (85.4)
Home state (%)**	South Australia	281 (38.1)	38 (34.9)	85 (34.0)	404 (36.8)
	Victoria	209 (28.3)	34 (31.2)	79 (31.6)	322 (29.4)
	New South Wales	102 (13.8)	15 (13.8)	53 (21.2)	170 (15.5)
	Queensland	42 (5.7)	10 (9.2)	12 (4.8)	64 (5.8)
	Western Australia	48 (6.5)	5 (4.6)	6 (2.4)	59 (5.4)
	Australian Capital Territory	18 (2.4)	2 (1.8)	7 (2.8)	27 (2.5)
	Tasmania	14 (1.9)	2 (1.8)	3 (1.2)	19 (1.7)
	Northern Territory	5 (0.7)	2 (1.8)	0 (0)	7 (0.6)
Family member worked in health profession		422 (57.2)	63 (57.8)	133 (53.2)	618 (56.3)
	Mother***	225 (53.3)	39 (61.9)	87 (65.4)	351 (56.8)
	Father***	144 (34.1)	28 (44.4)	51 (38.3)	223 (36.1)
	Other relative eg Aunt, Uncle***	126 (29.9)	17 (27.0)	44 (33.1)	187 (30.3)
	Sibling***	75 (17.8)	13 (20.6)	18 (13.5)	106 (17.2)

* Rurality is defined as having lived rurally for ≥8 years.

** Home State based on address on initial application.

***Includes only those who selected yes to primary question and is multiple response.

### Amount of preparation

**Figure 1:**
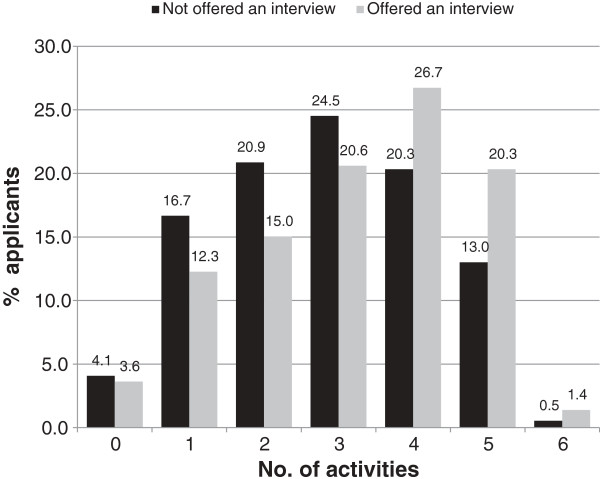
Number of preparation activities for UMAT by outcome.

**Figure 2:**
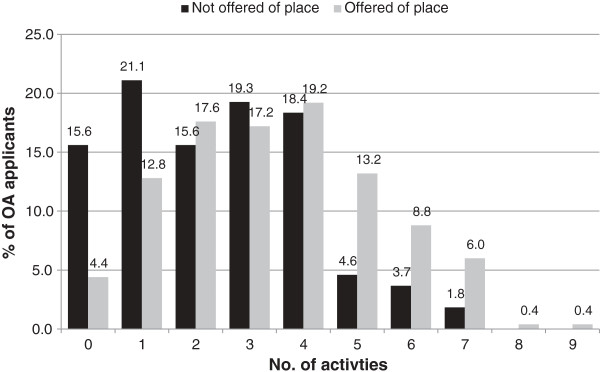
Number of preparation activities for oral assessment by outcome.

The number of preparatory activities undertaken for the UMAT and oral assessment component of the application process by the outcome (offered or not offered an oral assessment interview or offered or not offered a place) is shown in Figure 1 and Figure 2, respectively. The mean number of activities for the UMAT was 2.9 (2.8 for those who did not receive an offer of oral assessment interview and 3.2 activities for those who received an offer of an oral assessment interview). Regarding the UMAT, for every one additional activity that applicants undertook, the odds of being offered an interview increased by 1.22 (95% confidence interval [CI] 1.11 - 1.33; P < 0.001). The mean number of activities undertaken for the oral assessment was 3.1 ( 2.4 for those who did not receive an offer of a place and 3.4 for those who did receive an offer of a place). For the oral assessment, for every one additional activity that applicants undertook, they increased their odds of being offered a place by 1.36 (95% CI 1.19 - 1.55; P < 0.001).

### Type of preparation

#### UMAT

The 1097 applicants undertook a range of preparatory activities for the UMAT and the most common activity was the completion of example questions (83%) (Table 3). This was followed by familiarisation with the process (67%), speaking with others who had completed the UMAT previously (57%) and use of online services of private organisations (48%) (Table 3). Only a very small proportion of applicants indicated that they undertook no preparation for the UMAT (3%).

**Table 3:** UMAT and Oral assessment preparatory activities which resulted in the offer of an interview or a place in the medical school

**Stage of application process**	**Activity**	**Frequency of responses (%)**	**Odds ratio of being offered an interview or place given they did activity (95% CIs)**	**Uncorrected P value**	**Corrected P value**
UMAT (n = 1097)	Completed example questions	912 (83.1)	1.15 (0.81-1.61)	0.435	1
	Familiarised myself with the process	734 (66.9)	1.52 (1.15-2.00)	0.003	0.021*
	Spoke with people who had completed it before	621 (56.6)	1.16 (0.90-1.50)	0.254	1
	Utilised online services of private organisation	524 (47.8)	1.58 (1.23-2.04)	<0.001	<0.001*
	Attended training course offered by private organisation	395 (36.0)	1.75 (1.35-2.27)	<0.001	<0.001*
	Other (eg books, seminars)	45 (4.1)	1.14 (0.61–2.13)	0.680	1
	Nothing	37 (3.4)	0.75 (0.36-1.58)	0.454	1
	**Activity**	**Frequency of responses (%)**	**Odds ratio of being offered an interview or place given they did activity (95% CIs**)	**Uncorrected P value**	**Corrected P value**
Oral assessment (n = 37)	Learnt about the course structure	233 (64.9)	2.05 (1.29-3.26)	0.002	0.022**
	Discussed the oral assessment with previous applicants	211 (58.8)	1.72 (1.09-2.71)	0.019	0.209
	Prepared and learnt answers to possible questions	184 (51.3)	1.60 (1.02-2.52)	0.042	0.462
	Practiced interviews with family and friends	175 (48.8)	1.31 (0.83-2.06)	0.238	1
	Used the online services of a private company	86 (24.0)	2.06 (1.15-3.71)	0.014	0.154
	Attended a training course offered by a private company	60 (16.7)	1.24 (0.67-2.31)	0.493	1
	Refined and learnt a personal resume	57 (15.9)	9.73 (2.97-31.88)	<0.001	<0.001**
	School organised practice interviews	39 (10.9)	1.52 (0.69-3.31)	0.294	1
	Old scholars who studied/study medicine came to my school to talk	37 (10.3)	3.99 (1.38-11.56)	0.006	0.066
	Other (eg career advisor, self-reflection)	5858 (16.2)	1.17 (0.63-2.19)	0.617	1
	Nothing	27 (7.5)	1.04 (0.44-2.45)	0.920	1

*Statistically significant P values after Bonferroni correction for 7 comparisons.

** Statistically significant P values after Bonferroni correction for 11 comparisons.

After adjustment for multiple comparisons, several preparatory activities undertaken for the UMAT were significantly associated with obtaining an offer of an oral assessment interview. Those applicants who attended training courses by private organisations (OR 1.75, 95% CI 1.35 - 2.27; P < 0.001 corrected), used the online services of private organisation (OR 1.58, 95% CI 1.23 - 2.04; P < 0.001 corrected) or who familiarised themselves with the process (OR 1.52, 95% CI 1.15 - 2.00; P = 0.021 corrected) were significantly more likely to receive an offer of an interview than those who did not undertake the activity (Table 3).

#### Oral assessment

For the 359 applicants who were awarded an oral assessment interview, the most common preparatory activities were learning about the course structure (65%), discussing the process with previous applicants (59%), preparing answers for possible questions (51%) and having practice interviews with family and friends (49%) (Table 3). A small number of applicants (8%) did no preparation for the interview.

Two preparatory activities for the oral assessment interview were significantly associated with being offered a place in medical school after adjustment for multiple comparisons (Table 3). These activities were refining and learning a personal resume (OR 9.73, 95% CI 2.97 - 31.88; P < 0.001 corrected) and learning the course structure (OR 2.05, 95% CI 1.29 - 3.26; P = 0.022 corrected).

### Difficulties encountered

#### UMAT

Applicants (n = 1097) reported a number of difficulties with the UMAT. Time limit of the test (56%), inability to convey personal skills (50%) and the inability to determine if answers were correct (43%) were the most common difficulties encountered (Table 4).

**Table 4:** Difficulties encountered with the UMAT and Oral assessment and their association with either the offer of an interview or a place in the medical school

**Stage of application process**	**Difficulty**	**Frequency of responses (%)**	**Odds ratio of being offered an interview or place given they found the variable difficult (95% CIs)**	**Uncorrected P value**	**Corrected P value**
UMAT (n = 1097)	Time limit	619 (56.4)	0.88 (0.68-1.14)	0.325	1
	Can’t convey personal skills	547 (49.9)	0.67 (0.52-0.86)	0.002	0.026
	No idea if answers are correct	474 (43.2)	0.95 (0.74-1.22)	0.689	1
	Inability to prepare/learn for this type of test	364 (33.2)	0.47 (0.35-0.63)	<0.001	<0.001**
	Preparation costs	313 (28.5)	0.76 (0.57-1.01)	0.056	0.728
	Timing of UMAT in terms of school exams	293 (26.7)	0.48 (0.35-0.66)	<0.001	<0.001**
	Absence of breaks	194 (17.7)	1.10 (0.80-1.53)	0.554	1
	Attendance costs	200 (18.2)	0.68 (0.48-0.95)	0.025	0.325
	Exam environment	171 (15.6)	0.91 (0.64-1.29)	0.596	1
	Timing of results in terms of school exams	153 (14.0)	0.69 (0.47-1.02)	0.061	0.793
	Getting to the exam	94 (8.6)	0.86 (0.54-1.37)	0.527	1
	Other (eg stress, organisation)	124 (11.3)	0.93 (0.63-1.40)	0.752	1
	No difficulties	40 (3.7)	2.35 (1.25-4.43)	0.007	0.091
**Stage of application process**	**Difficulty**	**Frequency of responses (%)**	**Odds ratio of being offered an interview or place given they found the variable difficult (95% CIs)**	**Uncorrected P value**	**Corrected P value**
Oral Assessment (n = 359)	Hard to judge my performance	236 (65.7)	0.87 (0.54-1.41)	0.571	1
	Cost of attending interview	124 (34.5)	0.78 (0.49-1.24)	0.294	1
	It was difficult to structure answers	92 (25.6)	0.67 (0.40-1.10)	0.111	1
	Getting to the interview	62 (17.3)	0.75 (0.42-1.34)	0.334	1
	Questions were baffling	59 (16.4)	0.58 (0.32-1.03)	0.060	1
	Inability to learn/prepare for the interview	59 (16.4)	0.49 (0.27-0.86)	0.012	0.204
	Situational cards	53 (14.8)	1.59 (0.80-3.15)	0.186	1
	Absence of questions concerning personal achievements	51 (14.2)	0.95 (0.50-1.79)	0.862	1
	Interview environment	38 (10.6)	0.94 (0.45-1.94)	0.862	1
	Time pressure	24 (6.7)	1.33 (0.51-3.45)	0.554	1
	Timing of the interview	21 (5.9)	0.56 (0.23-1.37)	0.199	1
	The interviewer tried to distract me	20 (5.6)	1.79 (0.59-5.55)	0.299	1
	Received short notice of interview	15 (4.2)	0.48 (0.17-1.36)	0.249*	1
	Preparation costs	14 (3.9)	0.57 (0.19-1.68)	0.374*	1
	Presence of other applicants’ parents	11 (3.1)	0.76 (0.22-2.64)	0.741*	1
	Other (eg anxiety, intimidating interviewers)	58 (16.2)	1.17 (0.63-2.19)	0.617	1
	No difficulties	27 (7.5)	1.04 (0.44-2.45)	0.920	

*Fisher’s exact test was used.

** Statistically significant P values after Bonferroni correction for 13 comparisons.

The reported difficulties which resulted in a statistically significant decrease in the likelihood of being offered an oral assessment interview included difficulties with the timing of the UMAT in terms of school exams (OR 0.48, 95% CI 0.35 - 0.66; P < 0.001 corrected), the perceived inability to prepare or learn for this type of test (OR 0.47, 95% CI 0.35 - 0.63; P < 0.001 corrected) or feeling the test did not allow applicants to convey personal skills (OR 0.67, 95% CI 0.52 - 0.86; P = 0.026 corrected) (Table 4).

#### Oral assessment

Those who participated in the oral assessment interview (n = 359) also encountered several difficulties, the most common being their inability to judge their performance (66%), the cost of attending the interview (36%) and difficulty in structuring answers (26%) (Table 4). None of the difficulties identified with the oral assessment interview significantly decreased the likelihood of being offered a place in the program (Table 4).

## Discussion

This study is one of the first to investigate the impact of preparatory activities undertaken by applicants on the outcome of an application to medical school. Our results show that the more preparatory activities an applicant undertakes for the various stages of the selection process the more likely they are to be offered an interview or place in the medical school. We also found that certain distinct preparatory activities increased the odds of being offered an interview or a place. Some of the difficulties encountered during the process reduced the odds of being offered an interview.

There is limited research on the preparatory activities undertaken for medical school selection and there are even fewer studies that have investigated the impact of these activities on outcome (ie selection). Most of the literature on the role of preparation and coaching on performance in medical school exams comes from the USA. This research focuses on exams that occur once the student is in medical school, such as the USMLE step 1 exam [26,27], and often involve relatively small samples.[27] While there is some research on applicant preferences for new processes such as the Multiple Mini Interview (MMI) over traditional approaches [28,29], we found only one other study that had investigated student attitudes to parts of the selection process [30]. Their results correspond with the difficulties outlined in our study such as cost and perceived level of difficulty but only included those who had been successful in the medical school selection process. One small Australian study of 287 applicants assessed the impact of coaching on the UMAT and coaching and repeat testing on the MMI [21]. They found that coaching had a small effect on the parts of the UMAT test but was ineffective in improving the scores of the MMI. A small New Zealand study [24] investigated the impact of preparatory course and tutoring on the UMAT score and found these had no significant impact on the score. This contrasts with our results, although this study looked at the UMAT score result not success. However, they found that students undertaking the preparatory courses and spending more money on UMAT preparation had greater confidence in gaining an approved UMAT score.

Preparation for the various stages of the application process seems to contribute to a successful outcome and these results raise some important issues that have implications for the selection processes used by medical schools. We provide evidence that when there is a highly desired outcome [31], such as entry to medical school, applicants are likely highly motivated and consequently undertake a range of activities to ensure they achieve the desired outcome.

Of particular importance is the influence that preparatory activities have on this outcome. This particularly relates to the aptitude test such as the UMAT and UKCAT which are designed to measure innate aptitude and be less amenable to coaching. Messick and Jungeblut [32] defined ‘coaching’ as encompassing activities such as test familiarisation, practice with feedback, training strategies for specific formats, general test taking and skill development exercises. The majority of the preparatory activities identified by applicants in our study fall under this definition and for the UMAT, coaching does in fact seem to make a difference to the outcome. Research from the US, where coaching for the MCAT is long established, provides some evidence of practice effects, but the results are mixed and may be influenced by applicant motivation [33].

While the above issues are important, the most critical concern that this study raises is around equity. This is of particular importance in a climate where medical schools are increasingly seen as having a social accountability mandate. Prideaux et al [8] argue that widening access to unrepresented groups such as those from a rural, ethnic or low socio-economic background, is a values issue and not a ‘a technical question of choosing one selection method over another’ (page 219). We believe our results challenge this argument. If coaching in its broadest definition is associated with successfully applying to medicine, it has equity implications. The activities identified by the applicants in this study may not be accessible to all applicants due to cost, geography or opportunities. Training programs provided by commercial companies for medical school selection are costly, with basic packages start at $AU395 and range up to more than $AU1500. There can also be travel costs associated with attending workshops or courses. Some applicants may not be able to attend training if they live in rural or remote areas. The opportunity to discuss the application process with previous applicants may also favour applicants from medical families or those who attended private schools where access to old scholars to discuss the process or practice techniques is provided. Applicants from disadvantaged groups may not have access to such resources. These issues are of particular concern in a time when many medical schools/governments want to broaden the diversity of medical students, particularly from underrepresented groups [34]. This aspect may explain why, even with advent of strategies to increase underrepresented groups, widening access has not been totally successful [34]. Research has indicated that certain demographic characteristics are associated with a successful outcome to medical school [19]. Our results suggest that investigating the impact of socio-demographic characteristics on preparatory activities would be worthwhile in order to understand the role of these activities on outcome.

What can be done to address this additional variable in the selection process? Options to level the playing field may be the provision of coaching activities to disadvantaged groups, using processes shown to be less influenced by coaching such as the MMI, scores on such tests be weighted accordingly to account for level of disadvantage [35] or new selection processes such as the use of personality testing and testing emotional intelligence. However, changing the selection process is complicated and costly and it is likely that if a new process is developed, applicants will again adapt and find ways to increase their likelihood of being selected.

### Limitations

A key strength of the study is that data were collected as soon as the selection process had been completed when the applicants’ experiences were relatively fresh and it included applicants who were successful and unsuccessful, providing a full perspective on the selection processes. However, there are several limitations. The response rate achieved was 51% of the total applicants for 2007 and together with the fact that participants were drawn from one cohort may limit the generalisation of the results. It is likely that the results are biased to successful applicants, with the response rate lowest for those who were unsuccessful and highest for those who were successful.

While it was possible to identify individual preparatory activities or difficulties encountered that impacted on the outcome, we could not determine which combination of activities or difficulties that had the most influence on the outcome. This resulted from the large number of combinations possible as well as multiple combinations. Increasing the sample size or limiting the variables available for the respondents could address this issue in the future. Finally, while the results showed that certain preparatory activities were associated with a successful outcome, this does not indicate that the applicant is more suitable for the medical program or a career in medicine.

## Conclusions

Medical schools make an enormous effort to undertake a selection process that is fair and equitable and which selects students most appropriate for medical school and the course they provide. Our results indicate that performance in the selection processes can be improved by training. However, if these preparatory activities may be limited to those who can access them, the playing field is not even and increasing equity of access to medical schools will not be achieved.

## Competing interests

The authors declare that they have no competing interests.

## Authors’ contributions

CL conceived the idea, developed the design, coordinated the study, contributed to the analysis and interpretation of the results and drafted the manuscript. DT and IZ contributed to planning the research, analysis and the interpretation of the results and critically revised the paper. ML performed the analysis, contributed to the interpretation of the results and critically revised the paper. KS contributed to the planning of the research and critically revised the paper. All authors approved the final version of the manuscript.

## Pre-publication history

The pre-publication history for this paper can be accessed here:

http://www.biomedcentral.com/1472-6920/13/159/prepub

## Supplementary Material

Additional file 1 Survey questions relating to the preparatory activities and difficulties with the UMAT and Oral Assessment. Click here for file
