# Supplementary material for: The impact of preparatory activities on medical school selection outcomes: a cross-sectional survey of applicants to the university of Adelaide medical school in 2007
Source: BMC Med Educ. 2013 Dec 1;13:159. doi: 10.1186/1472-6920-13-159 (PMC4220562; doi:10.1186/1472-6920-13-159)
Supplement: Additional file 1 — Survey questions relating to the preparatory activities and difficulties with the UMAT and Oral Assessment. [file 1472-6920-13-159-S1.docx]

**Additional files 1: Survey questions relating to the preparatory activities and difficulties with the UMAT and Oral Assessment.**

**ABOUT THE UNDERGRADUATE MEDICINE ADMISSIONS TEST (UMAT):**

**1.** What did you do to prepare for the UMAT? *(tick any that apply)*

a. Completed example questions

b. Utilised online services of private companies such as MedEntry, Icarus etc.

c. Attended a face to face training course offered by a private company (i.e., MedEntry or Icarus)

d. Spoke with people who had done the UMAT in previous years

e. Familiarised yourself with the UMAT process (i.e., number of questions, time limit, location)

f. Other (specify)__________________________

g. Nothing →

**2.** What did you find difficult about the UMAT? *(tick any that apply).*

a. Difficult to convey personal skills via multiple choice exam questions

b. Time limit

c. Exam environment (size of room etc)

d. Inability to learn/prepare for this type of test

e. No idea whether answers were right or wrong

f. Absence of breaks between sections

g. Timing of the UMAT in terms of school exams

h. Timing of the release of UMAT results in terms of school exams

i. Getting to the exam

j. Costs related to UMAT preparation

k. Costs related to attending the UMAT

l. Other (specify)___________________________

m. Nothing

**ABOUT THE ORAL ASSESSMENT:**

**3.** What things did you do to prepare for the Oral Assessment at the

University of Adelaide? *(tick any that apply).*

⁮ a. School provided me with practice interviews

⁮ b. Practice interviews with friends/family

⁮ c. Utilised online services of private companies such as MedEntry, Icarus etc.

⁮ d. Attended a face to face training course offered by a private company (either 1 or 2 day)

⁮ e. Refined and learnt a personal resume

⁮ f. Discussed the Oral Assessment with people who had experienced it in previous years

⁮ g. Old scholars came to school and gave advice

⁮ h. Thought about possible questions and prepared and learnt answers

⁮ i. Learnt details concerning the course at the University of Adelaide

⁮ j. Other (specify)___________________________

⁮ k. Nothing →

**4.** What did you find difficult about the Oral Assessment at the University of Adelaide? *(tick any that apply).*

⁮ a. Received short notice of interview

⁮ b. Questions were baffling

⁮ c. Use of situational cards

⁮ d. Absence of questions about my personal achievements

⁮ e. Time pressure

⁮ f. Difficult to structure answers

⁮ g. Inability to prepare for the Oral Assessment

⁮ h. Hard to judge my performance

⁮ i. Environment of the Oral Assessment

⁮ j. Timing of the Oral Assessment

⁮ k. Getting to the Oral Assessment

⁮ l. Cost of travelling to the Oral Assessment

⁮ m. Costs related to preparing for the Oral Assessment

⁮ n. Presence of other applicant’s parents was intimidating

⁮ o. Interviewers tried to distract me

⁮ p. Other (please specify)_____________________

⁮ q. Nothing
